# S-adenosyl methionine synthetase SAMS-5 mediates dietary restriction-induced longevity in *Caenorhabditis elegans*

**DOI:** 10.1371/journal.pone.0241455

**Published:** 2020-11-11

**Authors:** Chia-Chang Chen, Chiao Yin Lim, Pin-Jung Lee, Ao-Lin Hsu, Tsui-Ting Ching

**Affiliations:** 1 Institute of Biopharmaceutical Sciences, National Yang-Ming University, Taipei, Taiwan; 2 Institute of Biochemistry and Molecular Biology, National Yang-Ming University, Taipei, Taiwan; 3 Taiwan International Graduate Program in Molecular Medicine, National Yang-Ming University and Academia Sinica, Taipei, Taiwan; 4 Research Center for Healthy Aging, China Medical University, Taichung, Taiwan; 5 Division of Geriatric and Palliative Medicine, Department of Internal Medicine, University of Michigan, Ann Arbor, MI, United States of America; University of Hong Kong, HONG KONG

## Abstract

S-adenosyl methionine synthetase (SAMS) catalyzes the biosynthesis of S-adenosyl methionine (SAM), which serves as a universal methyl group donor for numerous biochemical reactions. Previous studies have clearly demonstrated that SAMS-1, a *C*. *elegans* homolog of mammalian SAMS, is critical for dietary restriction (DR)-induced longevity in *Caenorhabditis elegans*. In addition to SAMS-1, three other SAMS paralogs have been identified in *C*. *elegans*. However, their roles in longevity regulation have never been explored. Here, we show that depletion of *sams-5*, but not *sams-3* or *sams-4*, can extend lifespan in worms. However, the phenotypes and expression pattern of *sams-5* are distinct from *sams-1*, suggesting that these two SAMSs might regulate DR-induced longevity via different mechanisms. Through the genetic epistasis analysis, we have identified that *sams-5* is required for DR-induced longevity in a *pha-4/FOXA* dependent manner.

## Introduction

Dietary restriction (DR) is the most robust intervention that extends lifespan across species [1–4]. In *Caenorhabditis elegans*, *sams-1* is a key regulator in DR-induced longevity identified from a RNAi screen for longevity genes [5]. RNAi knockdown of *sams-1* increases the lifespan of wild-type animals by 21%, but fails to further extend the lifespan of *eat-2 (ad1116)* mutants [5]. Besides, *sams-1*-mediated lifespan extension is also independent of *daf-16/*FOXO transcription factor [5]. Furthermore, mRNA expression of *sams-1* is significantly reduced in *eat-2(ad1116)* mutants.

*sams-1* encodes an evolutionarily conserved enzyme, S-adenosylmethionine synthetase (SAMS), which is also known as the methionine adenosyltransferase (MAT) in mammals. SAMSs are required for survival of all living organisms. They are the only enzymes that can catalyze the formation of an essential coenzyme, S-adenosyl methionine (SAM) from ATP and methionine [6]. SAM is linked to many key biological processes. The best known and the most important one is transmethylation, since most of the SAM generated per day is used in transmethylation reactions under normal conditions in mammals [7]. In transmethylation reactions, SAM donates its methyl group to a large variety of acceptors in reactions catalyzed by various methyltransferases. In addition to acting as the principal methyl group donor, SAM is also known as the precursor for polyamine biosynthesis [8, 9], and a precursor of glutathione (GSH) through transsulfuration [10].

In mammals, *MAT1A* and *MAT2A* encode for the MAT catalytic subunits, <1 and <2 [11]. *MAT1A* is expressed mainly in the adult liver, whereas *MAT2A* is widely distributed. In the liver, which is the major site of the biosynthesis and metabolism of SAM in mammals, up to 50% of the daily uptake of methionine is converted to SAM by SAMS [12]. In *C*. *elegans*, *sams-1* has been recognized as the orthologs of *MAT1A* and three other genes, *sams-3*, *sams-4*, *and sams-5*, have been designated as the orthologs of *MAT2A*. Unlike *sams-1*, the biological functions of the other SAMS paralogs are mostly unknown. In this study, we have demonstrated that *sams-5*, but not *sams-3* or *sams-4*, is also involved in longevity regulation by DR. However, we found that animals lacking *sams-5* do not display the phenotypic characteristics that were found in *sams-1* mutants, suggesting that *sams-1* and *sams-5* might have different functions and regulate DR-induced longevity via distinct mechanisms in *C*. *elegans*. Furthermore, we found that the FOXA transcription factor PHA-4 is required for *sams-5* to modulate lifespan.

## Materials and methods

### *C*. *elegans* strains used in the study

DA1116: *eat-2(ad1116)II*, CF1037: *daf-16(mu86)I*, EQ153: *sams-1(ok2946)*, EQ159: *sams-5(gk147)V*, EQ355: *iqEX105[sams-5p*::*sam-5*::*gfp +rol-6]*, EQ1021: *eat-2(ad1116)II*; *iqEX105[sams-5p*::*sam-5*::*gfp +rol-6]*. DA1116, CF1037 and wild-type *Caenorhabditis elegans* (N2) strains were obtained from the Caenorhabditis Genetic Center at the University of Minnesota. Worms were maintained and handled as described previously [13, 14].

### Lifespan analysis in *C*. *elegans*

Lifespan analyses were conducted at 20°C as described previously [15–17]. At least 72 animals were used for each experiment. The viability of the worms was scored every two days. In all experiments, the pre-fertile period of adulthood was used as day 0 for lifespan analysis. Strains were grown at 20°C for at least two generations before use in lifespan analysis. Survival plots, *p* values (Log-Rank), and proportional hazards were determined using Stata12. (Stata Crop) software.

### RNA-interference (RNAi) experiments

The identity of all RNAi clones was verified by sequencing the inserts using M13-forward primer. All the clones were from Julie Ahringer’s RNAi library. HT115 bacteria transformed with RNAi vectors expressing dsRNA of the genes of interest were grown at 37°C in LB with 10 μg/ml tetracycline and 50 μg/ml carbenicillin, then seeded onto NG-carbenicillin plates and supplemented with 100 μl 0.1M IPTG. Eggs were added to plates and transferred to new plates every 2–5 days.

### Plasmid and transgenic animal constructions

Transgenic strains were generated by microinjection. 2 kb *sams-5* promoter and *sams-5* insert fragments were cloned into pPD117.01 GFP expression vector. SAMS-5::GFP fusion constructs were injected at 20 ng/μl along with the *rol-6* co-injection marker (pRF4) at 100 ng/μl. Differential interference contrast microscopy and fluorescence images were acquired with an Olympus BX63 microscope.

### Oil red O staining for lipid content

Age-synchronized worms were collected and washed with M9 buffer. Worms were then fixed in 2**x** MRWB buffer (160 mM KCl, 40 mM NaCl, 14 mM EGTA, 1 mM spermidine-HCl, 0.4 mM spermine, 30 mM PIPES [piperazine-N,N′-bis(2-ethanesulfonic acid); pH 7.4], 0.2% ß-mercaptoethanol) with 2% paraformaldehyde at room temperature for one hour. The worms washed with M9 buffer, then dehydrated in 60% isopropanol for 15 min at room temperature and stained with 60% Oil Red O solution at room temperature overnight. Animals were mounted on 2% agarose pads and imaged at 10x magnification using an OLYMPUS BX63 with DIC using Olympus Microsuite software. Oil red O intensities were determined using ImageJ software (NIH).

## Results

### Depletion of *sams-5*, but not *sams-3* or *sams-4*, extends lifespan in *C*. *elegans*

**Fig 1:**
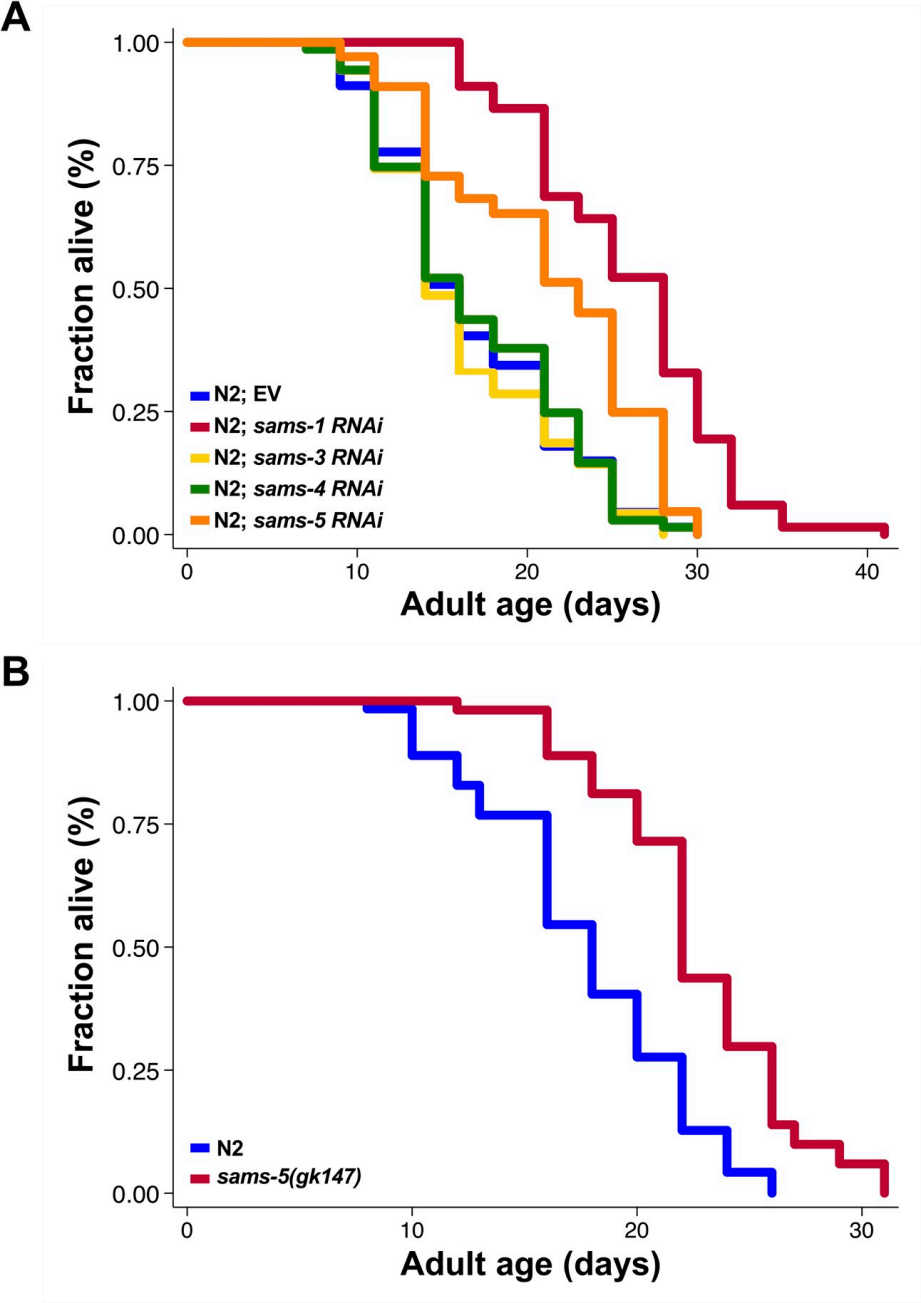
Loss of *sams-5* induces longevity in *C*. *elegans*. **A)** Lifespan analysis of wild-type (N2) worms grown on control (blue), *sams-1*(red), *sams-3 (yellow)*, *sams-4* (green) or *sams-5* (orange) RNAi bacteria. **B)** Lifespan analysis of wild-type N2 animals (blue) and *sams-5(gk145)* mutants (red). Additional lifespan replicates are included in S1 Table.

In mammals, while displaying distinct expression patterns, both *MAT1A* and *MAT2A* are involved in SAM biogenesis. Since the amino acid sequences of four SAMS paralogs in *C*. *elegans* are highly homogeneous, we would like to further investigate whether *sams-3*, *sams-4*, or *sams-5* is also involved in longevity regulation. We thus performed lifespan analysis on wild-type worms grown on *sams-1*, *sams-3*, *sams-4*, or *sams-5 RNAi* bacteria, respectively. Besides *sams-1*, we found that RNAi knockdown of *sams-5* could also markedly extend wild types’ lifespan by 25% (Fig 1A). We have also confirmed that *sams-5* RNAi was not cross-reacted with *sams-1* (S1A Fig). Interestingly, unlike what we have observed in *sams-1* and *sams-5* depletion animals, RNAi knockdown of *sams-3* and *sams-4* did not affect the lifespan of wild-type animals (Figs 1A and S1B).

### SAMS-1 and SAMS-5 are S-adenosylmethionine synthetases with distinct physiological roles

**Fig 2:**
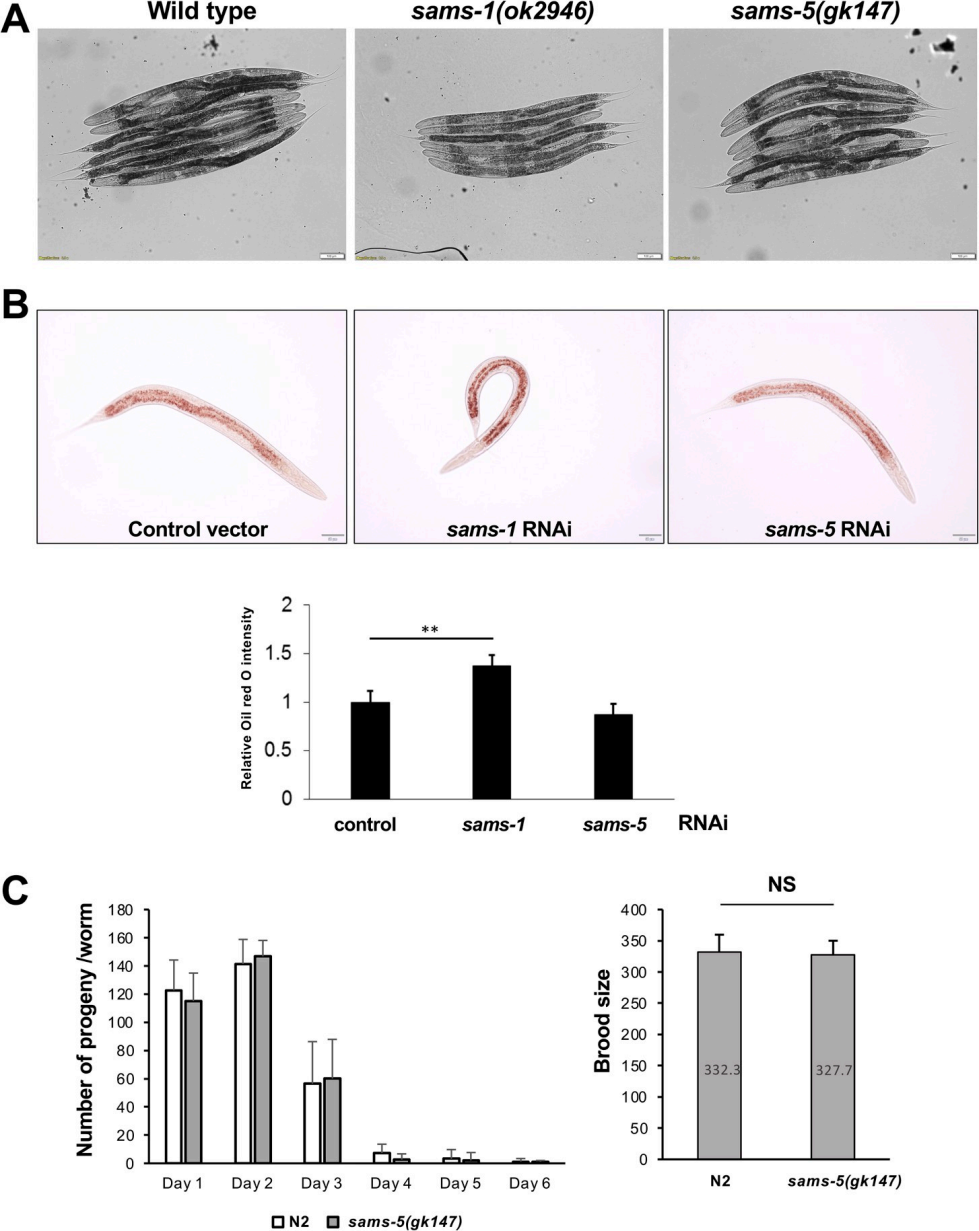
Comparison of phenotypes between *sams-1* and *sams-5* mutants. **A)** Images of Day 1 adults of wild type animals, *sams-1* mutants, and *sams-5* mutants. Scale bar, 100 μm. **B)** Oil red O staining was applied to L4 N2 wild type animals treated with control vector, *sams-1* RNAi, and *sams-5* RNAi. Scale bar, 50 μm. **C)** Reproduction timing and **D)** Brood sizes of wild type worms and *sams-5(gk147)* mutants (n = 10).

It has been reported that RNAi knockdown of *sams-1* results in reduced body sizes and causes a significant accumulation of lipid droplets in the intestine [18, 19], which is the *C*. *elegans* counterpart to the gut, *liver*, and adipose tissue. Thus, we first compared the appearance of N2 wild type, *sams-1(ok2946)* and *sams-5(gk147)* animals at Day 1 of adulthood. We found that the body size of *sams-5* mutants, unlike *sams-1* mutants, is not significantly different from the wild-type worms (Fig 2A). We then further confirmed that the levels of intestinal fat were not affected by depletion of *sams-5* by Oil red O staining in L4 worms (Fig 2B). Previous studies have also indicated that *sams-1* RNAi results in reduced brood size and slightly delays reproduction timing [20]. However, both the brood size and reproduction timing are not significantly affected in *sams-5* mutants (Fig 2C). Together, we presume that *sams-1* and *sams-5* might exert distinct physiological roles in *C*. *elegans*.

### SAMS-5 exhibits different expression patterns in the larval and adult stages

**Fig 3:**
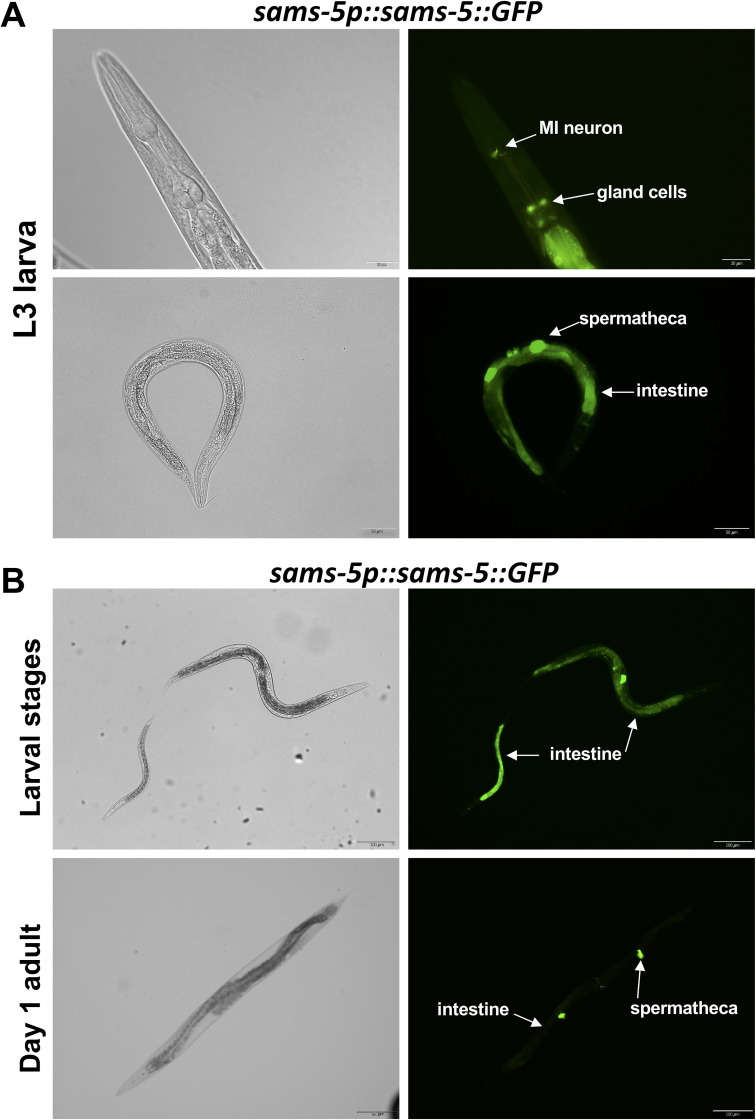
The expression patterns of SAMS-5. **A)** Images of transgenic larvae carrying *sams-5p*::*sams-5*::*gfp*. Scale bar: Upper panel, 20 μm; lower panel, 50 μm. **B)** The expression of SAMS-5 in the larval stages and Day 1 adults. Various cells and organs are indicated by white arrows. Scale bar, 100 μm.

To gain more insights into the location of activity and the role of *sams-5*, we generated transgenic mutants carrying a *sams-5*::*gfp* transgene. This transgene is driven by 2 kb of genomic sequence upstream of the endogenous *sams-5* gene and expresses a protein reporter that fuses GFP to the C-terminus of SAMS-5. *Nakano et al*. have previously demonstrated that *sams-5* is expressed in the unpaired MI neuron [21]. Indeed, we observed a strong expression of SAMS-5 in pharyngeal MI neuron. In addition to the MI neuron, SAMS-5::GFP was also found in the gland cells, the intestine, and spermatheca during the larval stages (Fig 3A and 3B). However, when worms reach adulthood, the intestinal SAMS-5::GFP was markedly reduced, while SAMS-5::GFP in other cells remains present (Fig 3B).

### *sams-5* contributes to DR-induced longevity via *pha-4/FOXA* transcription factor

**Fig 4:**
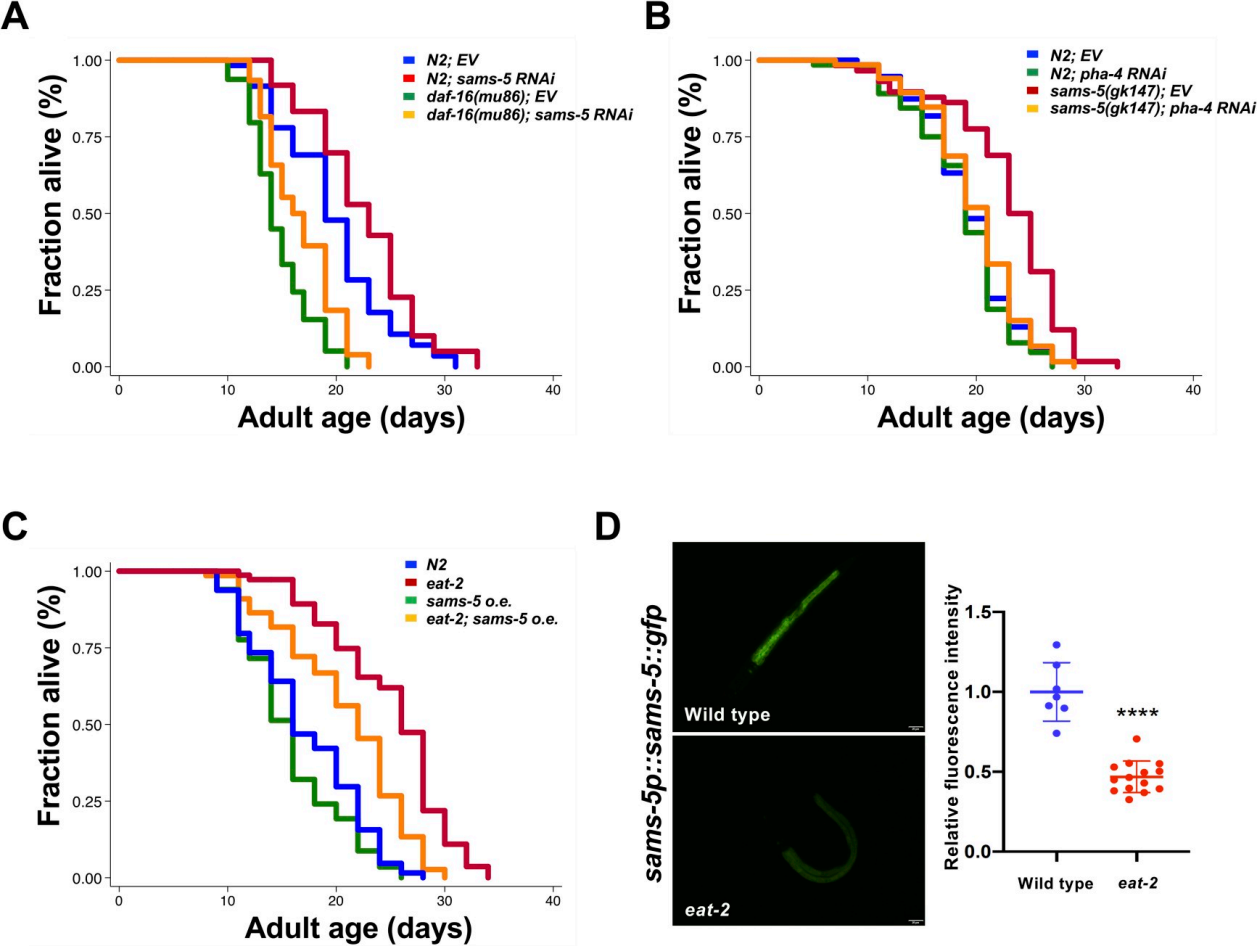
Lifespan extension in *sams-5* mutants is independent of *daf-16*, but dependent on *pha-4*. Lifespan analysis of **A)** wild-type N2 animals (blue and red) and *daf-16(mu86)* worms (green and orange) grown on control or *sams-5* RNAi bacteria. **B)** wild-type N2 animals (blue and green) and *sams-5(gk147)* mutants (red and orange) grown on control or *pha-4* RNAi bacteria from L4 stage. **C)** wild type N2 animals (blue), *sams-5* overexpressing mutants (green), *eat-2* mutants (red), and *eat-2;sams-5* overexpressing mutants (orange). Additional lifespan replicates are included in S1 Table. **D)** Images of L1/L2 wild type N2 animals or *eat-2* mutants expressing *sams-5p*::*sams-5*::*gfp* (left). Scale bar, 20 μm. Quantification of *sams-5-*::*gfp* expression (right).

Next, we further explored the mechanisms by which *sams-5* regulates longevity. DAF-16, a FOXO transcription factor, is the master regulator for insulin/insulin-like growth factor (IGF-1) signaling (IIS) and signals from the reproductive system to extend lifespan in *C*. *elegans*. Thus, we first tested the role of *daf-16* in *sams-5*-mediated longevity. We found that *sams-5* RNAi knockdown could still extend lifespan in *daf-16(mu86)* null mutants (Fig 4A), suggesting that *daf-16* is not required for *sams-5* to increase longevity. We then examined whether *sams-5*-mediated longevity is dependent on *pha-4/FOXA* transcription factor, another known regulator of longevity. We performed lifespan analysis on N2 wild-type worms and long-lived *sams-5(gk147)* mutants fed with control or *pha-4* RNAi bacteria from L4 stage. As shown in Fig 4B, RNAi depletion of *pha-4* completely abolished the long-lived phenotype of *sams-5(gk147)* mutants, indicating that *sams-5*-mediated longevity is dependent on *pha-4/FOXA*.

Since PHA-4/FOXA has been shown to be critical in mediating the longevity effects of DR [22], we then investigated whether *sams-5* is involved in the regulation of DR-induced longevity. To do so, we generated transgenic animals carrying additional copies of *sams-5* gene to determine whether over-expression of *sams-5* is sufficient to suppress the longevity effect of DR. We found that the lifespan extension in *eat-2(ad1116)* mutants, a well-known genetic model of DR [23], is markedly suppressed by the over-expression of SAMS-5 (Fig 4C). Furthermore, the level of SAMS-5 protein were significantly reduced in *eat-2* mutants (Fig 4D). Taken together, our findings suggest that *sams-5* might act upstream of PHA-4 to mediate the DR-induced longevity.

## Discussion

While both *sams-1* and *sams-5* are clearly involved in mediating DR-induced longevity, it appears that they may do so via distinct mechanisms. For example, *sams-1* is also known for its role in lipid homeostasis. The depletion of *sams-1* elevates SBP-1-dependent lipogenesis through decreasing phosphatidylcholine synthesis, leading to a significant increase in lipid droplet size and lipid content in the intestinal cells [19]. However, we found that *sams-5* mutants do not display such phenotype found in *sams-1* mutants (Fig 3A and 3B). Similarly, *sams-5* mutants do not display reduced and delayed reproduction phenotype, which is also typical in *sams-1* mutants [24]. Furthermore, by generating SAMS-5::GFP transgenic animals, we found that the expression patterns of *sams-5* are different from *sams-1* [24]. *sams-1* is found to be expressed mainly in the intestine, body-wall muscle, and hypodermis, whereas *sams-5* is constantly expressed in the MI neuron, gland cells, and spermatheca (Fig 3).

Intriguingly, *sams-5* could be found in the intestine only during larva stages but not in adults. It is known that *MAT2A*, the mammalian homolog of *sams-5*, is the predominant isozyme of MAT in the fetal liver. *MAT2A* is later replaced by *MAT1A*, the homolog of *sams-1*, in the adult liver [25]. It is interesting that similar MAT isozyme switching during development is present in both the nematode and mammals, indicating that the expression of the two MATs might be regulated through an evolutionarily conserved mechanism.

Although the molecular and cellular mechanism by which SAMS-1 and SAMS-5 mediates DR-induced longevity might be different, the common denominator of the two is the altered SAM/SAH level resulted from modulating SAMS-1 or SAMS-5 as the major molecular function of SAMS is to produce SAM. SAM is widely involved in many different cellular processes such as methylation of DNA, RNA, proteins, phospholipids, hormones, and neurotransmitters in mammals [26]. Dietary methionine deficiency could limit the production of SAM and result in a decreased SAM/SAH level, which in turn inhibits the methylation of various methyl group acceptors [27]. Interestingly, reducing dietary methionine levels (methionine restriction, MR) has been shown to increase the lifespan of flies, mice [28], and rats [29–31]. In *C*. *elegans*, *Cabreiro et al*. have reported that, by altering bacterial methionine metabolism, metformin induces methionine restriction and extends lifespan in worms [32]. Furthermore, the long-lived *sams-1* mutants have a 65% decrease in SAM levels [19]. Taken together, altering SAM level might be a common cellular strategy to regulate aging and longevity. Thus, further defining the roles of different SAMSs and SAM-dependent methylation reactions in the context of DR could significantly enrich our understanding on the regulation of aging and longevity.

## Supporting information

S1 Table Statistical data for *C*. *elegans* lifespan experiments. (PDF)

S1 Fig A) Relative mRNA expression of sams-1 (white) and sams-5 (black) in wild type N2 animals treated with control vector, sams-1 and sams-5 RNAi, respectively. B) RNAi knockdown efficiency of sams-3 and sams-4. (mean ± S.D.) The experiments were repeated three times and the significance of difference was determined by one-way ANOVA relative to control and indicated by asterisks (**p < .01, ****p < .0001).
(PDF)
