# Supplementary material for: S-adenosyl methionine synthetase SAMS-5 mediates dietary restriction-induced longevity in Caenorhabditis elegans
Source: PLoS One. 2020 Nov 11;15(11):e0241455. doi: 10.1371/journal.pone.0241455 (PMC7657561; doi:10.1371/journal.pone.0241455)
Supplement: S1 Table — (PDF) [file pone.0241455.s001.pdf]

S1 Table. Statistical data for *C. elegans* lifespan experiments

strain name	treatment	mean	p (Log-rank test)	%change	75%	n	
N2 (WT)	EV	16.8 ± 0.33			21	67/71	Figure 1A
N2 (WT)	*sams-1 RNAi*	26.0 ± 0.21	<0.0001	54.2	20	67/76	
N2 (WT)	*sams-3 RNAi*	16.4 ± 0.28	0.6338		21	70/75	
N2 (WT)	*sams-4 RNAi*	17.1 ± 0.40	0.8607		21	70/74	
N2 (WT)	*sams-5 RNAi*	21.2 ± 0.39	<0.0001	26.0	25	65/73	
N2 (WT)		15.6 ± 0.82			18	59/72	
sams-5(gk147)		21.5 ± 1.08	<0.0001	37.6	26	61/72	
N2 (WT)		17.9 ± 1.38			22	56/72	Figure 1B
sams-5(gk147)		21.8 ± 0.91	<0.0001	21.8	26	56/72	
N2 (WT)	EV	18.2 ± 0.84			21	65/72	
N2 (WT)	*sams-5 RNAi*	20.4 ± 0.75	0.0074	12.1	23	62/72	
N2 (WT)	EV	18.5 ± 0.87			22	57/72	
N2 (WT)	*sams-5 RNAi*	21.5 ± 0.04	0.0002	16.2	24	47/72	
N2 (WT)		19.6 ± 0.40			23	63/72	
sams-5 OE		19.6 ± 0.30	0.9047		23	56/72	
N2 (WT)	EV	16.4 ± 0.69			19	69/72	
N2 (WT)	*sams-5 RNAi*	17.9 ± 0.44	0.0192	10.1	21	64/72	
daf-16(mu86)	EV	14.9 ± 0.81			18	54/72	
daf-16(mu86)	*sams-5 RNAi*	17.4 ± 0.36	<0.0001 (vs *daf-16*)	16.8	19	63/72	
N2 (WT)	EV	19.7 ± 0.81			19	69/72	Figure 4A
N2 (WT)	*sams-5 RNAi*	22.5 ± 1.13	0.009	14.2	21	64/72	
daf-16(mu86)	EV	14.7 ± 0.31			18	54/72	
daf-16(mu86)	*sams-5 RNAi*	16.9 ± 0.73	<0.0001 (vs *daf-16*)	15.0	19	63/72	
N2 (WT)	EV	19.2 ± 0.71			22	67/72	
N2 (WT)	*sams-5 RNAi*	20.9 ± 0.84	0.0207	8.9	24	48/72	
N2 (WT)	*pha-4 RNAi (L4)*	17.1 ± 0.58	0.0041	-10.9	21	63/72	
N2 (WT)	*sams-5 RNAi; pha-4 RNAi(L4)*	17.9 ± 0.47	0.0515	-6.8	21	57/72	
N2 (WT)	EV	19.3 ± 0.66			22	67/72	Figure 4B
N2 (WT)	*sams-5 RNAi*	23.1 ± 0.57	<0.0001	19.7	24	48/72	
N2 (WT)	*pha-4 RNAi (L4)*	18.7 ± 0.67	0.0206	-3.1	21	63/72	
N2 (WT)	*sams-5 RNAi; pha-4 RNAi(L4)*	19.9 ± 0.68	0.0616		21	57/72	
sams-5(gk147)	EV	23.6 ± 0.67	<0.0001 (vs N2)	22.3	27	58/72	
sams-5(gk147)	*pha-4 RNAi (L4)*	19.9 ± 0.69	0.3759 (vs N2)		23	61/72	
N2		17.2 ± 1.14			22	64/72	Figure 4C
eat-2		25.0 ± 0.88	<0.0001	45.4	28	33/96	
sams-5 OE		15.9 ± 0.59	0.105		18	63/72	
eat-2; sams-5 OE		20.9 ± 1.08	0.0002	21.5	26	40/96	
N2		19.8 ± 0.69			24	61/72	
eat-2		23.0 ± 0.62	<0.0001	16.2	26	37/72	
eat-2; sams-5 OE		18.3 ± 2.12	0.7584		24	42/72	
N2		18.7 ± 0.77			22	54/72	
eat-2		24.8 ± 0.33	<0.0001	32.6	28	58/96	
eat-2; sams-5 OE		21.1 ± 1.05	0.0061	12.8	30	27/72	
