# Supplementary material for: S-adenosyl methionine synthetase SAMS-5 mediates dietary restriction-induced longevity in Caenorhabditis elegans
Source: PLoS One. 2020 Nov 11;15(11):e0241455. doi: 10.1371/journal.pone.0241455 (PMC7657561; doi:10.1371/journal.pone.0241455)
Supplement: S1 Fig — A) Relative mRNA expression of sams-1 (white) and sams-5 (black) in wild type N2 animals treated with control vector, sams-1 and sams-5 RNAi, respectively. B) RNAi knockdown efficiency of sams-3 and sams-4. (mean ± S.D.) The experiments were repeated three times and the significance of difference was determined by one-way ANOVA relative to control and indicated by asterisks (**p < .01, ****p < .0001). (PDF) [file pone.0241455.s002.pdf]

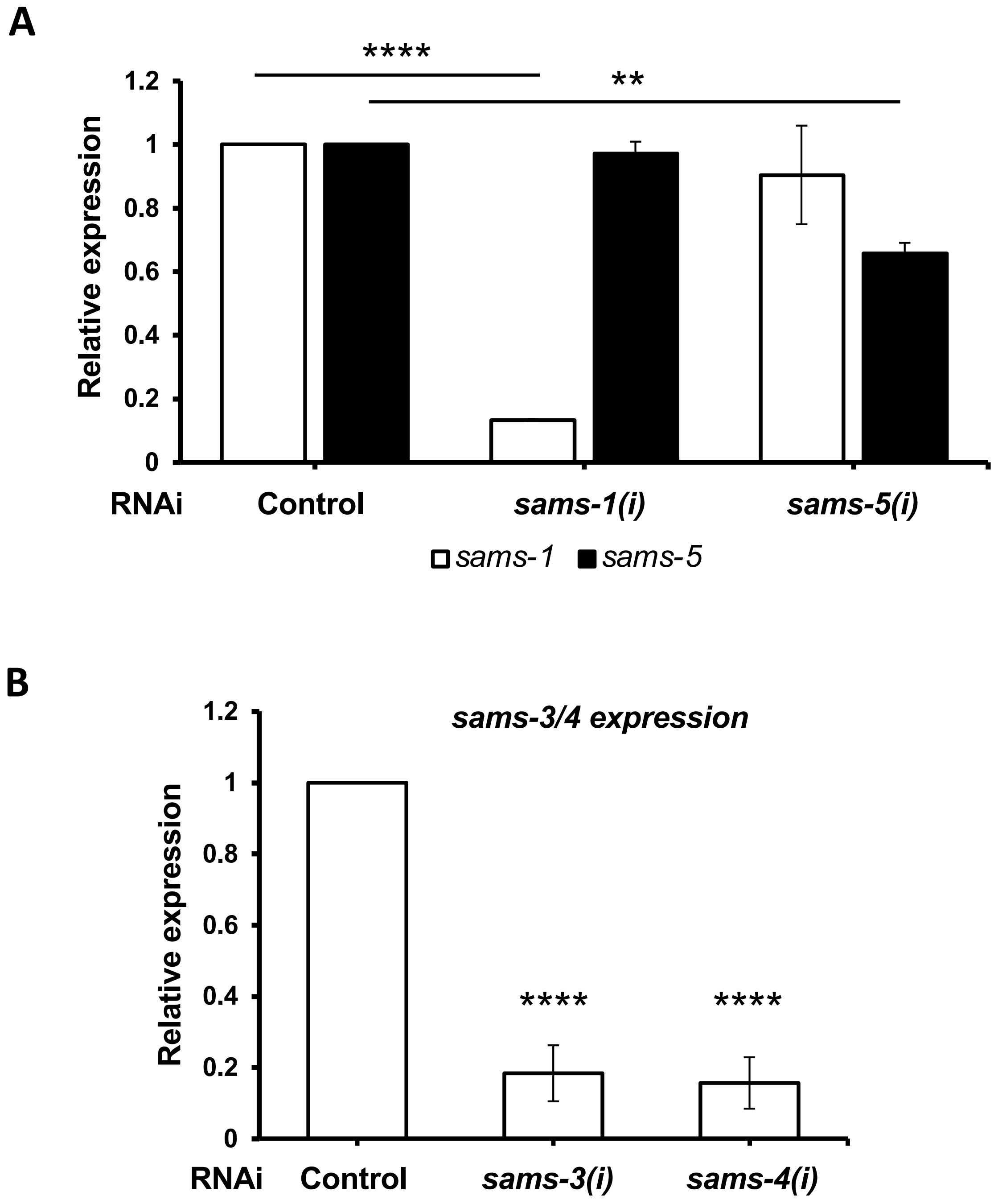

S1 Fig.

A) Relative mRNA expression of *sams-1* (white) and *sams-5* (black) in wild type N2 animals treated with control vector, *sams-1* and *sams-5* RNAi, respectively. **B)** RNAi kncokdown efficiency of *sams-3* and *sams-4.* (mean ± S.D.) The experiments were repeated three times and the significance of difference was determined by one-way ANOVA relative to control and indicated by asterisks (**p < .01, ****p < .0001).
